# The Community-Engaged Lab: A Case-Study Introduction for Developmental Science

**DOI:** 10.3389/fpsyg.2021.715914

**Published:** 2021-08-19

**Authors:** Judy Liu, Scott Partington, Yeonju Suh, Zoe Finiasz, Teresa Flanagan, Deanna Kocher, Richard Kiely, Michelle Kortenaar, Tamar Kushnir

**Affiliations:** ^1^Department of Human Development, Cornell University, Ithaca, NY, United States; ^2^Office of Engagement Initiatives, Cornell University, Ithaca, NY, United States; ^3^Sciencenter, Ithaca, NY, United States

**Keywords:** COVID-19, community engagement, online developmental science, citizen science, research-community partnerships, broader impacts

## Abstract

Due to the closing of campuses, museums, and other public spaces during the pandemic, the typical avenues for recruitment, partnership, and dissemination are now unavailable to developmental labs. In this paper, we show how a shift in perspective has impacted our lab's ability to successfully transition to virtual work during the COVID-19 shut-down. This begins by recognizing that any lab that relies on local communities to engage in human research is *itself a community organization*. From this, we introduce a *community-engaged lab* model, and explain how it works using our own activities during the pandemic as an example. To begin, we introduce the vocabulary of mission-driven community organizations and show how we applied the key ideas of mission, vision, and culture to discussions of our own lab's identity. We contrast the community-engaged lab model with a traditional bi-directional model of recruitment *from* and dissemination *to* communities and describe how the community-engaged model can be used to reframe these and other ordinary lab activities. Our activities during the pandemic serve as a case study: we formed new community partnerships, engaged with child “citizen-scientists” in online research, and opened new avenues of virtual programming. One year later, we see modest but quantifiable impact of this approach: a return to pre-pandemic diversity in our samples, new engagement opportunities for trainees, and new sustainable partnerships. We end by discussing the promise and limitations of the community-engaged lab model for the future of developmental research.

## Introduction

Developmental science does not happen in a vacuum. Our science crucially depends on our relationship with local communities—and in particular the organizations and spaces where children and families live, work, and play. This includes schools, museums, daycare centers, churches, playgrounds, local businesses, and the many community non-profits that serve children's and families' interests. It is standard practice in our discipline to engage with local organizations when we recruit families to participate in research. It is also standard to include them as part of our plans to disseminate research beyond academic publications. Our impact and success depend on cultivating collaborative research partnerships with our local communities.

We believe research labs can benefit from more explicitly acknowledging this fact. In this paper, we use our own lab as a case study, and argue that a simple shift in perspective can have a positive impact on research, dissemination, and bridge-building between labs and local communities. Our own shift to this perspective began prior to the 2020 coronavirus pandemic, and we believe it allowed us to transition to pandemic-era work with relative ease.

We begin by describing the principles of mission-driven community organizations and how they can be used to create a new model for developmental science labs. We then describe the model of the *community-engaged lab* and contrast it with the standard bi-directional model. Using examples, we demonstrate how this model enabled us to pivot to new recruitment methods, programming, and dissemination in a completely virtual pandemic-era lab. We then present evidence suggesting that community engagement *works*: internal records show that our virtual engagement efforts helped to maintain a representative participant pool on par with our recruitment pre-pandemic, that we have increased opportunities for early-career researchers to get involved in public-facing programs and outreach, and that we have expanded to include engagement with new and different types of community partners. We conclude with discussion of the unique benefits that engagement can provide to our communities and our science, as well as some ways that in-person and digital avenues of community engagement can complement each other in the future.

## Principles of Mission-Driven Community Organizations

Community organizations that work with children and families define their purpose and contributions with a *mission, vision*, and *values* (Crutchfield and Grant, 2007). The *mission* is an explanation of how the organization's vision will be accomplished, while the *vision* is a statement that describes long-term goals or purpose. A strong mission statement is rooted in the present and is also purpose-driven: it is future-oriented and often a means of achieving a greater vision. A mission statement also typically includes a target audience, the organization's contribution, and factors that distinguish it from other organizations. *Values* are fundamental guiding principles and beliefs that help define an organization's identity and an organization's culture. An effective value statement explicitly states how members of an organization are expected to act toward fellow internal members as well as how the organization will treat the community as a whole.

Successful organizations make their mission, vision, and values explicit (Crutchfield and Grant, 2007). Though we had begun some of these discussions prior to the pandemic, this topic took priority in our discussions when the shut-down occurred. How does a lab like ours develop a mission statement? Our lab conducts basic research in cognitive and social development. It is a place where future scientists are trained, at the undergraduate and graduate level, by participating in the day-to-day work of conducting research, by studying developmental theory, and by collaborating and exchanging ideas. Thus, it is perhaps obvious that our mission centers around research, teaching, and mentorship. Essential target audiences therefore include students that receive training and mentorship in the lab and the scientific community that we reach through scholarly publications.

Does our mission extend beyond the research, teaching, and mentorship goals of our scientific enterprise? It does if our vision does. Our research program centers around early childhood learning, cognitive, and social-cognitive development from a constructivist perspective. Moreover, for many years, we have been in partnerships with educational institutions—including our local science museum, local schools, and youth programs. By combining these, our explicit statement of vision became: “to empower communities with a holistic understanding of child development, so that every child can actively explore and learn about the world around them, supported by caring adult guidance and the surrounding culture.” This statement reflects what we believe to be fundamental principles of early learning and development, and also reflects what our community partnerships have taught *us* about *their* missions, and our respective contributions as partners.

As is true of other community organizations, our mission and vision are carried out through daily actions, guided by shared values. Again through discussion, we worked to make our values explicit. What we settled on was a culture of *trust, collaboration*, and *acceptance* that defines how we interact within our lab and guides ethical action toward our participants, our partners, and others. In our view, trust is the foundation of responsible research conduct, protecting data integrity, and working in teams. Similarly, we view collaboration is the basis of creative scholarship, and involves a willingness to combine strengths, to teach and mentor, and to listen openly in a free exchange of ideas. Finally, acceptance allows expression of different perspectives, intellectual risk-taking, and appreciation of each other as we work toward common goals.

This internal culture informs our relationships with the local community. We trust each other to represent the lab honorably when interacting with participants and their families. Communities trust us to ask how we can best meet their needs and value their assets, and not assume that we know what those needs and assets are (Kretzmann and McKnight, 1993). Trainees in the lab *double as community ambassadors* as they engage in active volunteerism in community organizations and science communication. Several examples are found in sections below. Importantly, in our minds, this culture opens up space for new collaborations, for new ideas for programming, for grants, and for research.

We want to make clear that, at least for us, the importance of making these mission, vision, and value statements explicit was more about the process than the outcome (Ash and Clayton, 2009). Last March, the motivation to think in this way was made urgent by the complete fragmentation of everything that made us a lab prior to the pandemic. Individual students and researchers went home. Children were home. Parents were struggling to work, care for, and educate their children alone. We as a group of researchers were looking for a way to connect, to maintain our lab identity, and to have a shared experience in the virtual world that resembled the one we had when we were physically together.

We chose to use the language of mission-driven organizations as a tool to help us stay connected. Throughout meetings, discussions of our lab *identity*—what it was, how it was changing, what we could do to maintain it in the face of massive change—were a motivating force driving us to keep going. Prior to the pandemic, we thought of ourselves as part of two communities: A local community of organizations serving children and families, and a global network of labs dedicated to developmental research. In our discussions early in the pandemic, we felt strongly that we wanted to emphasize our role in the local community even more, and we wanted to participate in children's lives as they were radically changing.

We also want to make clear that there is no “right” way to approach making these statements explicit. For us, a useful starting point was to think about the research program of the lab, broadly construed, and to incorporate feedback from our closest community partners. We suggest beginning the process wherever it makes sense and being willing to see where it leads.

## Embedded in a Local Community

**Figure 1:**
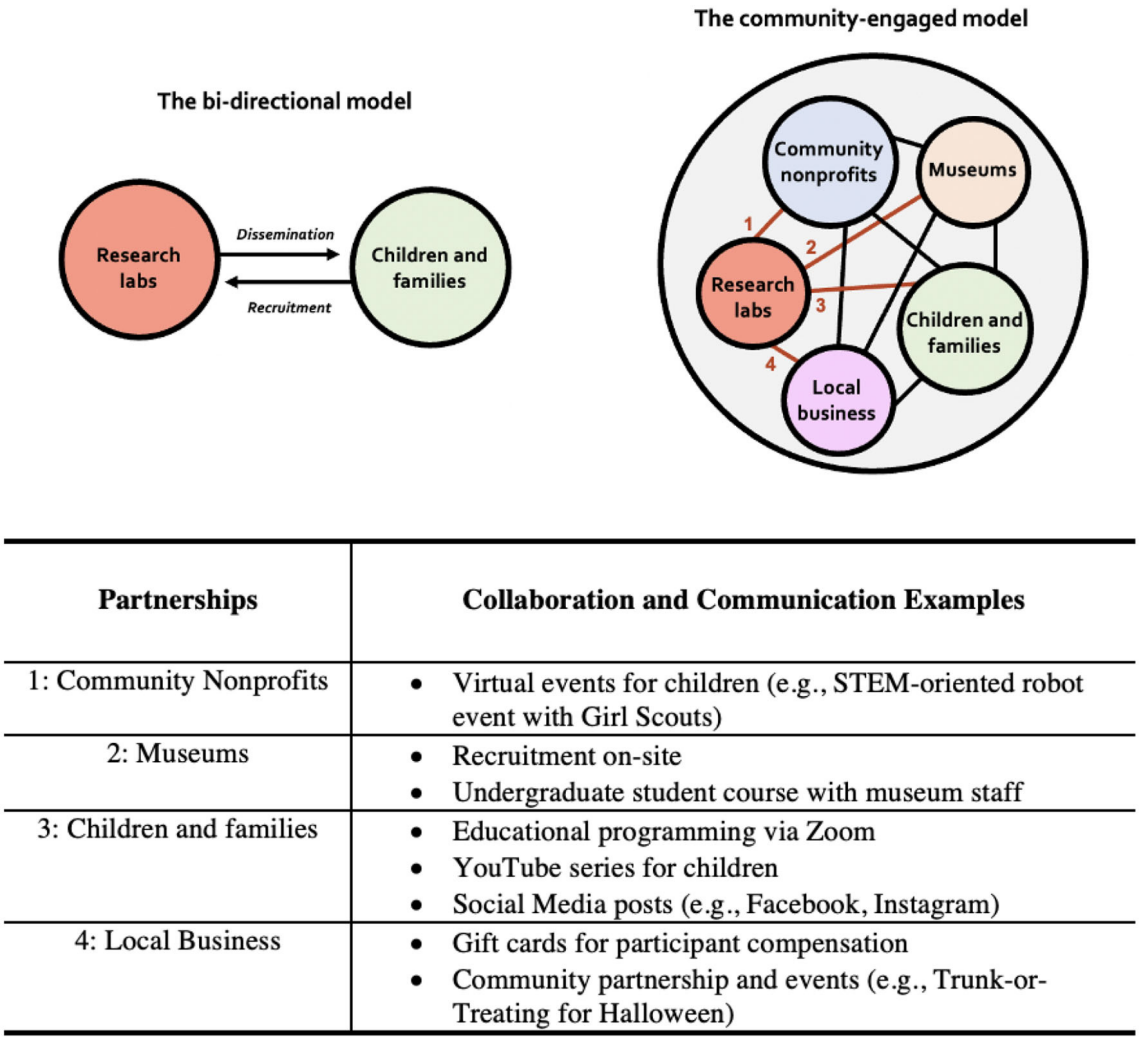
The schematic depiction of the differences between the traditional model (left) and the community-engaged model (right) with a list of collaboration and communication examples (bottom). Under the traditional model, the typical relationship between developmental research labs and community stakeholders is a bi-directional exchange whereby communities provide data to scientists provide knowledge to communities. In the community-engaged model, research labs' explicitly aticulate commitments to reinforce patnerships through resource and knowledge sharing based on reciprocity and mutual benefit (Kretzmann and McKnight, 1993).

Scientists are dedicated to creating knowledge, and often think of the infrastructure of their organization as merely a necessary means to this end. In this section, we examine the traditional lab practices involved in producing scholarship through the lens of a community-driven mission. Figure 1 shows a visual representation of the *community-engaged lab* model side-by-side with the traditional, bi-directional model. The traditional model depicts a bi-directional relationship between developmental science labs (which are members of universities and a broader network of scholars) and communities (either local or virtual) where children and families (our participants, who are also meant to be the beneficiaries of scientific knowledge) live. This bi-directional model is one that most developmental labs have in mind, at least implicitly, when they set up mechanisms for recruitment and dissemination. Our community-engaged model is different: developmental labs like ours and their scientific mission are *embedded* within a local community. Note that we do not mean this as a replacement for ways that labs connect with other communities, such as a global network of scholars, nor do we advocate operating separately from academic institutions in which labs reside. But rather, the community-engaged model's intent is to serve as a principled shift in guiding how we relate to local community organizations and the children and families which they serve. Instead of engaging with local communities from the outside, community-engaged developmental labs productively operate within, among, and alongside organizations that serve children and families in the local community.

Concretely, this model resulted in a reframing of many of our ordinary daily tasks—in particular, our public-facing activities. Recruitment and data collection are reframed under this model as *collaborative* actions: we grow our participant pool of child “citizen scientists” by supporting and sustaining long-term partnerships with other organizations whose mission, vision, and values align with ours. Dissemination of research findings is reframed as having the broader goal of science *communication*. In this way, all of our interactions with the community are opportunities for conveying our mission outwards. During the early days of the pandemic a top priority was to return, with minimal disruption, to our research. Our community partners—the science museum, local schools, and community centers which were our main avenues for reaching children and families prior to the shut-down—were experiencing their own upheavals. Our commitment to find new ways to stay engaged with the community impacted decisions we made as an organization about how to continue to work.

Below we describe how each reframing was put into action in our lab. For the purposes of illustration, the examples below are organized in two sections, with the recognition that the distinction is somewhat arbitrary. In the traditional bi-directional model, the dual-goals of recruitment and dissemination work together. In our community-engaged model, even as they are reframed, virtual collaborations and partnerships open up new opportunities for science communication. Efforts to engage in virtual science communication lead back toward goals of citizen science and toward samples of children that are more inclusive and representative of our local community.

Notably, the community-engaged lab model, at least in our case, was not intended as a change of direction away from our scientific mission, but rather a way of supporting it. Whether this approach has a long-term impact on our lab, on our trainees, or on our ability to do science, and whether it can be useful to other labs, remains to be seen. Despite this, we argue that for the success of our own pandemic-era work, our identity as a community-engaged lab was critical.

## Recruitment Reframed

**Figure 2:**
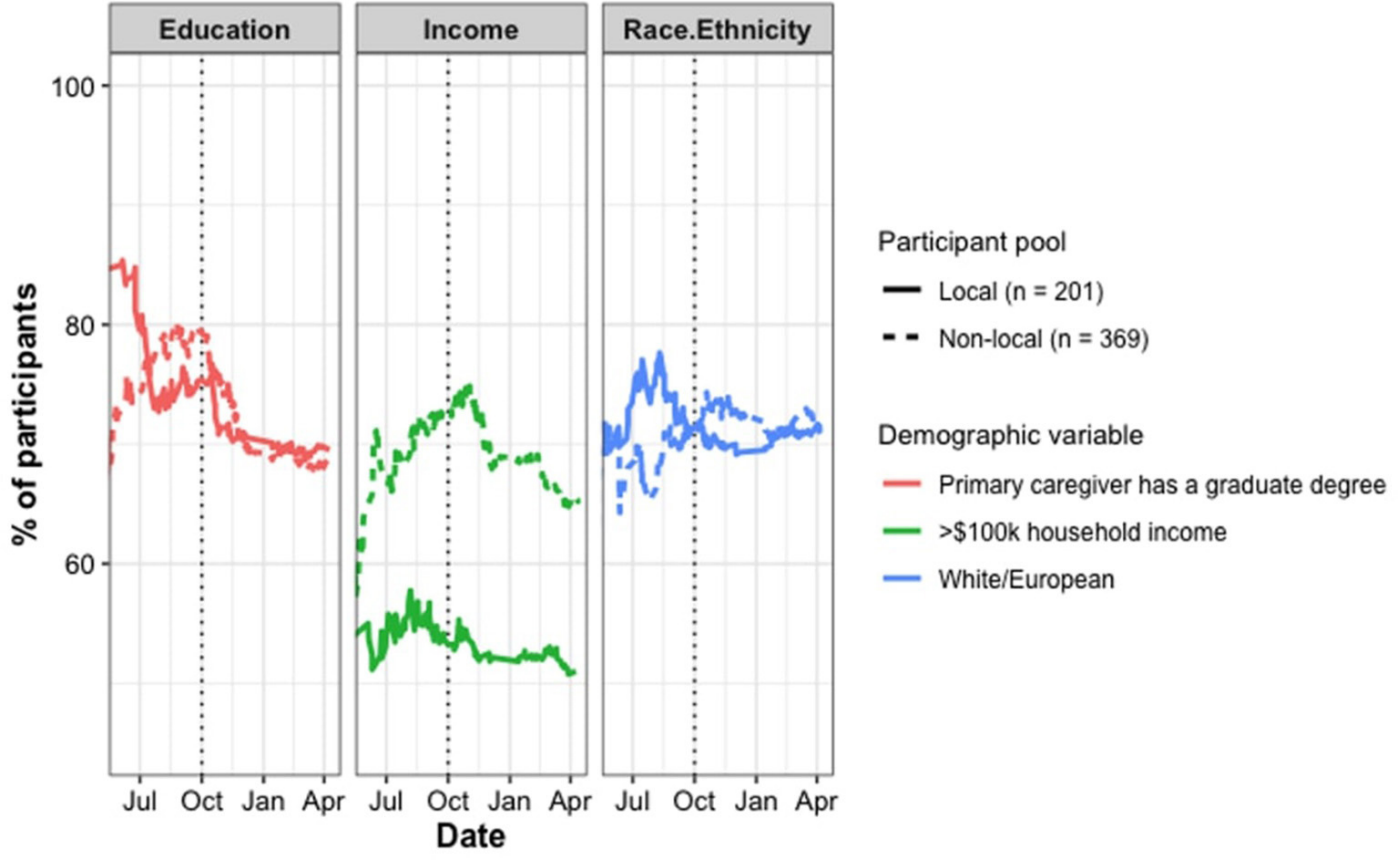
Demographic indicators among local and non-local participants (June 2020–April 2021). The time series graph show the percentage of participants (starting *n* = 30 local, *n* = 36 non-local) whose primary caregiver has a graduate degree (left, red), whose anual household income is greater than $100,000 (center, green), and who are white/European (right, blue). Solid lines indicate trends for local participants and dashed line indicate trends for non-local participants. High numbers on the y-axis indicate higher income, higher education, and perdominantly white samples. The checked vertical line signifies the date of our lab meeting in October 2020, when we set new goals for reaching more participants outside of academic families.

Under the traditional bi-directional model, recruitment *from* communities results in a supply of data *to* human-participant labs. One unintended consequence of this model of research participation is that it perpetuates the current predominance of homogeneous (predominantly white, predominantly high-SES) samples in research (Nielsen et al., 2017; Lourenco and Tasimi, 2020). Pre-pandemic, standard lab recruitment was successful for engaging with families that were already comfortable coming to labs, or those that had the time and resources to go to science and children's museums, or those that were familiar with (and trusting of) research protocols like signing consent forms. During the pandemic, these limitations were exacerbated by issues of availability of computers and stable internet connections, and the ability of parents to make time to schedule and connect through virtual lab visits—parents took on more roles as full-time caregivers, teachers for homeschooling, and, in some cases, employees working from home. Thus, it was no surprise that, initially, our lab (and perhaps others) saw samples becoming *less* representative of our local communities than they were before (see Figure 2).

We hoped that our community-engaged lab model could be a starting point for reaching groups of children and families that represent the diversity of our local community. In theory, this works by creating long-term sustainable collaborations with community partners, and establishing trust. But what about in the short term? Could this idea help us meet the immediate needs of functioning as a lab during the pandemic? Here we describe some examples, and signs of success.

### Participant Incentives and New Community Partnerships

Under the bi-directional model, labs that have the means to provide incentives operate with the idea that gifts or other types of compensation are exchanges based on single interactions. If, for example, a family comes into the lab to participate in a study, they may leave with a gift or monetary compensation. If a school or museum partners with the lab, they too may receive gifts or donations.

Recognizing the indirect effects that our incentives have on communities, and reframing with this in mind, has led us to turn gifts and other forms of compensation into opportunities for more engagement with our local economy. For example, behavioral research labs often use Amazon gift cards to compensate participants for their time because Amazon is a globally accessible and convenient option. However, such an approach sacrifices a valuable opportunity to work with the local community, and an ethical duty to ensure that gift cards are used by children. To align with our community-engaged mission, when our lab transitioned to virtual studies, we sought out community partnerships with child-focused local businesses. Several factors motivated this search: we wanted the gift cards to be used for children, wanted to give participants alternative options to support local businesses, and wanted to start building collaborative community partnerships with organizations that were both a part of our local economy and were mission-driven in the service of children and families.

We started by approaching a local toy store that was operating virtually via their website. In our initial meeting, we discussed our common interests in children's learning. Our exchange resulted in a collaboration that benefited the business as well as our research: we created a mechanism for distributing gift cards that participants could use online or in-store (once in-person retail became an option again). Our lab handled the creation of advertisements (i.e., posters for the store) and the creation of visually appealing coloring-pages to serve as gift cards. We also trained researchers on how to offer the gift-cards as an alternative to Amazon without appearing coercive, similar to how we train researchers in informed consent. After a 6 month pilot program with the toy store, we expanded. We approached a local bookstore with a particular interest in children's education and literacy with the same idea, and tailored the gift-cards to this business, using their preferred system for keeping track of gift cards, and different advertising strategies.

### Indicators of Success

It was informative for us to gather some data on the impact of our local business partnership programs. Of the 526 gift cards given out to all participants, local and remote, since we began online data collection, 429 (81.6%) requested Amazon gift cards and 97 (18.4%) requested local gift cards. Perhaps telling is that both local and remote participants requested local gift cards −21.6% (21/97) local gift cards went to participants outside of our county. Also telling is that local participants requested local gifts almost half the time −46% (76/165) requested local business gift cards rather than Amazon gift cards. We take this as a sign that many families were enthusiastic to support local businesses during the pandemic through our incentive program.

Of course, the goal of our relationship with both of these local businesses was not just to create alternative participant incentives, but also to build new community partnerships. Along these lines, we have maintained open discussions with each business with the idea that the collaboration could grow. Encouragingly, both business owners had multiple creative ideas about new ways to collaborate: the toy store suggested that we could help test out models of new toys that toy companies send to the store before they display it in store, and the bookstore was interested in hosting a book reading event for children. Although these events are planned, they are on hold until current pandemic restrictions are lifted, but we take the enthusiasm for continued collaboration as a concrete sign of success.

### Museum Collaborations and Living Labs

Science and children's museums are mission-driven community organizations whose vision and values align well with developmental labs. There is already a decades-long tradition of developmental labs collaborating with local children's museums using “living labs” to recruit participants and collect data, and to disseminate findings on the museum floor (Sobel and Jipson, 2015; Callanan et al., 2020). The benefits of these partnerships have been noted before: for labs, museums offer convenient access to children and families, and a greater chance of reaching more representative participant samples though this sample is still limited to the patrons of local museums. For families, museum research reduces barriers associated with travel to university labs, and increases opportunities to learn about developmental science and see it in action. For museums, the partnerships offer multiple benefits from positive visitor experience to opportunities for additional grant support.

Prior to the pandemic, our lab maintained such a partnership with our local science museum. We recruited and collected data on the museum floor in a living lab-style exhibit. We wrote and received several collaborative grants to support both organizations. We also designed and implemented several museum service-learning courses in which students worked on museum projects—ranging from designing exhibits and programs, to creating evaluation tools that could be used to measure impact.

The pandemic brought enormous challenges to museums, as they were forced to close to the public, furlough staff, and rethink their operations. From our perspective, it is tempting to view this as another example of how pre-pandemic ways of conducting research effectively shut down. But guided by our long-standing partnership, and by the community-engaged model that supported it, we take another view. On many fronts, members of our research team continued to engage with the museum and looked for ways to help support their work financially and logistically. In addition, the museum service-learning course ran virtually this spring, and included some in-person components. The museum project for students in spring 2021 was an evaluation of long-standing exhibits: students were assigned an exhibit to observe, conducted an evidence-based analysis of how the exhibit currently supports STEM learning, and, under the guidance of the instructor and museum staff, offered suggestions for improvements. Like its predecessor projects, this one was aligned with the training and mentorship missions of the lab, as well as the learning mission of the museum to create experiences for multi-age learning communities.

### Community Events

In addition to growing long-term partnerships, we sought opportunities to participate in community events. On Halloween, our local mall hosted a Trunk-or-Treat event where families drove by a row of socially distanced vendors and local businesses to receive Halloween treats. A team of students from our lab planned for the event by preparing bags of treats with small flyers to advertise our studies to families. The event was a success, as we reached ~270 families who inquired about lab research and participation. Importantly, too, it was an opportunity for lab members to engage in outreach and develop their leadership and communication skills.

Single events can sometimes lead to long-term partnerships. For example, members of our lab connected with the local Girl Scout leaders to arrange a virtual STEM-oriented event about child-centered robot design. The initial idea was just a single virtual session: an educational program used to illustrate how humans and robots can “collaborate” on tasks. Over 40 children ages 4 to 13 participated in a virtual activity which asked them to direct a person pretending to be a “robot” around an obstacle course set up in their space. Children who had someone to collaborate with were provided instructions and examples of how to give directions, and children who did not have someone helped direct a student “robot assistant” by giving directions over the conference call. The session concluded with an opportunity for Q&A about women in STEM and their career paths.

The event illustrates how the research and training goals of the lab can combine with addressing the needs of a community organization. From a research standpoint, the event was an adaptation of a recently published study which communicated the activity and research to the public. In addition, it exposed children to one of the major challenges in robotics: navigation in collaborative tasks. For training, it was an opportunity for our lab members to talk about their work and aspirations. For the community partner, they hoped to use the event to inspire and motivate girls in STEM careers. The troop leaders' feedback after the event was overwhelmingly positive, and they have been very receptive and enthusiastic about future events that allow researchers to gather observational data and convey principles of design thinking to young girls.

## Dissemination Reframed

Under the traditional bi-directional model, dissemination usually lives in the space of academic discourse, such as publications and conferences. Most labs also include some dissemination to local communities—such as websites and lab newsletters—but this communication is generally thought of as completely separate. This accepted practice of scientific vs. public dissemination reinforces the separation between research and the communities it ultimately serves. For instance, it takes 17 years on average for findings in scholarly journals to reach the general public (Trochim, 2010). Additionally, if and when research findings do become more available, accessibility emerges as another barrier due to the financial cost of journal subscriptions, the time needed to thoroughly comprehend the studies, and the lack of readability as a result of the density of research jargon.

Adopting a community-engaged lab model encouraged us to think about lab *communication* rather than dissemination. Under this reframing, any opportunity for exchanges of knowledge with the community carries equal value. During the pandemic, this translated into actionable steps: we created internal mechanisms to train lab members to be good science communicators, and we used the tools of the virtual environment to make science accessible whenever possible.

### Trainees as Community Ambassadors

Developmental labs commonly rely on young researchers-in-training to manage the many daily tasks involved in conducting human participant research with children and families. In the community-engaged lab model, trainees are not only a valuable resource to the lab, but a bridge between the lab and community in which it is embedded.

As the examples below illustrate, trainees in the community-engaged model become the most important resource for all public-facing communication activities. For us, it was important to ensure that each of these activities were free, inclusive, and accessible; we conceived them as low-stress ways for our lab members to interact with the public and encouraged each trainee to think of themselves as an ambassador of science in the community.

A lot of ideas for activities were driven by the creativity of student trainees, who were responsive to community feedback: lab members often came back from virtual (or in-person) events with comments and suggestions from the children and families, and the mutual exchange of knowledge and ideas motivated further activities. Training students to think more about diversity in research, informing them on how a lab can serve the community, and encouraging them to take action has long-term benefits. While not all student trainees are interested in a career in research, many of them are interested in working with children and families across multiple professions. This experience as community-engaged lab ambassadors will translate to leadership and service in their future.

### Exchanging Ideas for Communication and Outreach

To facilitate involvement of all lab members in communication and outreach, we devoted one meeting per month to discuss communication goals. The meetings were structured in the following way: At the beginning of the Fall 2020 semester, lab members chose to be part of one of three small teams: an *in-person team* that organized and participated in community events, an *outreach team* that focused on connecting with local community organizations, and a *social-media team* that advertised studies online to reach families outside of the local community. Each team nominated a student leader to ensure progress.

Acting in small teams (rather than as a larger group) leads to student trainees feeling heard in their ideas and having more ownership over their actions (Avey et al., 2009). Leaders and coordinators in the lab play an important role here: it is essential that these people genuinely understand the lab's mission of promoting diversity and serving as a community engaged organization, and to constantly reflect on and assess the communication goals of the lab. When leaders in the lab actively communicate these values of promoting diversity and serving as a community engaged organization, it encourages conversations among lab members and establishes these values as the lab's culture.

### Communication Activities

Below we describe some of the results of this process, and give examples of our communication activities over the last year:

1. **Children Doing “Citizen Science.”***Citizen science* is defined as “the collection and analysis of data relating to the natural world by members of the general public, typically as part of a collaborative project with professional scientists” (Oxford Languages, 2021). This idea motivates national recruitment efforts such as “Children Helping Science” which was organized by a consortium of labs in response to the pandemic shut-downs (Sheskin et al., 2020). Of course, the idea of citizen science is compatible with the bi-directional model as well. But under the community-engaged model, every time a child helps *us* as a “citizen scientist” is also an opportunity for *us* to engage in science communication. This was particularly important this year, as families were stretched to their limits and children were spending more time in front of screens (Richtel, 2021). Consistent with our effort to re-imagine the lab, we emphasized to trainees and families that participating in research is not just a transaction, but rather an opportunity to learn about, and actively participate in doing science. Individual participant interactions, then, were bookended by discussion of the study purpose, opportunities to ask questions, and follow up newsletters with updates and findings, and other ways to get involved with the lab. To this end, 52% (186 out of 361) of children participated more than once in our online studies.
2. **Educational Programming via Zoom**. Over the summer, we hosted a series of educational programs designed for children between the ages of 3- and 8-years-old. Once a week, members of the lab took turns hosting 20–30 min sessions during which children would participate in an active-learning experience. All of the topics were based on lab members' own interests and ideas, with the only restriction being that it would be fun for preschool and school-age kids. Topics included visual illusions, robots, how to grow a garden, yoga, karate, origami, games, crafts, and more. Typically, anywhere between 3 and 10 children would join each week, and we found that children would commonly come back for repeated visits or for studies as a result. These programs offered children and families an informal, playful introduction to our team. They also kept all of us connected to children and to each other during the difficult summer when most other research engagement opportunities for students were unavailable.
3. **YouTube Kid's Series**. The fall semester brought new challenges for families facing school on screens. In our early fall reflections, and with feedback from parents, we recognized that a change to accommodate family schedules was needed. From this we moved our programming asynchronously, in the form of weekly 3–5-min YouTube videos. Once again, the focus was on active learning, play, and curiosity. Motivated by our own passions and interests, we wanted to inspire children to try something new, (e.g., learn a magic trick, make animations) or investigate a fascinating and perhaps unexplored phenomenon in the world around them (e.g., why do the leaves change colors in the fall?). In total, 22 kids joined us across 7 sessions during the summer. To date, the 17 videos in the YouTube series have received a total of 645 views.
4. **Social Media**. Like many other developmental labs, we use social media to reach potential participants locally and across the country. In addition to study advertisements, we have followed the growing trend of using social media for science communication. There are already successful campaigns on social media that directly aim to educate parents and practitioners about the science of child development. We viewed our efforts as an opportunity for research trainees to apply knowledge from the classroom to solving a real-world problem. As a group, we were encouraged to reflect critically about our science education: Was there a way to translate what we learn in labs and classrooms to community-engagement? We used reflective prompts, questions like: “What is one thing you learned about child development that surprised you?” or “What research finding you've read inspires you?” We also talked about children's lives and how they had changed, and looked for media and scientific coverage on the changing roles of parents. We aimed to keep our posts light, fun, and grounded in our own experiences. Our focus is on communicating curiosity, being ourselves, showing support for communities, rather than delivering information.

## Community Engagement Works: Measuring Impact

Throughout this paper, we have emphasized how a simple shift in perspective—thinking of developmental labs as embedded within a network of local community organizations—can help engender a number of positive outcomes for local children, families, community partners, and early career trainees. Throughout this paper, we have shared anecdotal evidence of such impacts: we created two new, long-lasting community partnerships, we hosted educational events and weekly programming that together encouraged hundreds of local children to be active, curious “citizen scientists.” All along the way, early career trainees played a crucial role in fostering such relationships, and in turn gained valuable leadership skills.

In addition to this anecdotal evidence of impact, we also have internal data that shows the community-engaged lab model's role in making our online participant pool quantifiably more inclusive and representative of our local community. Next we discuss how our lab assessed and modified our community-engaged recruitment aims by analyzing the standard demographic information collected from our study consent forms.

**Figure 3:**
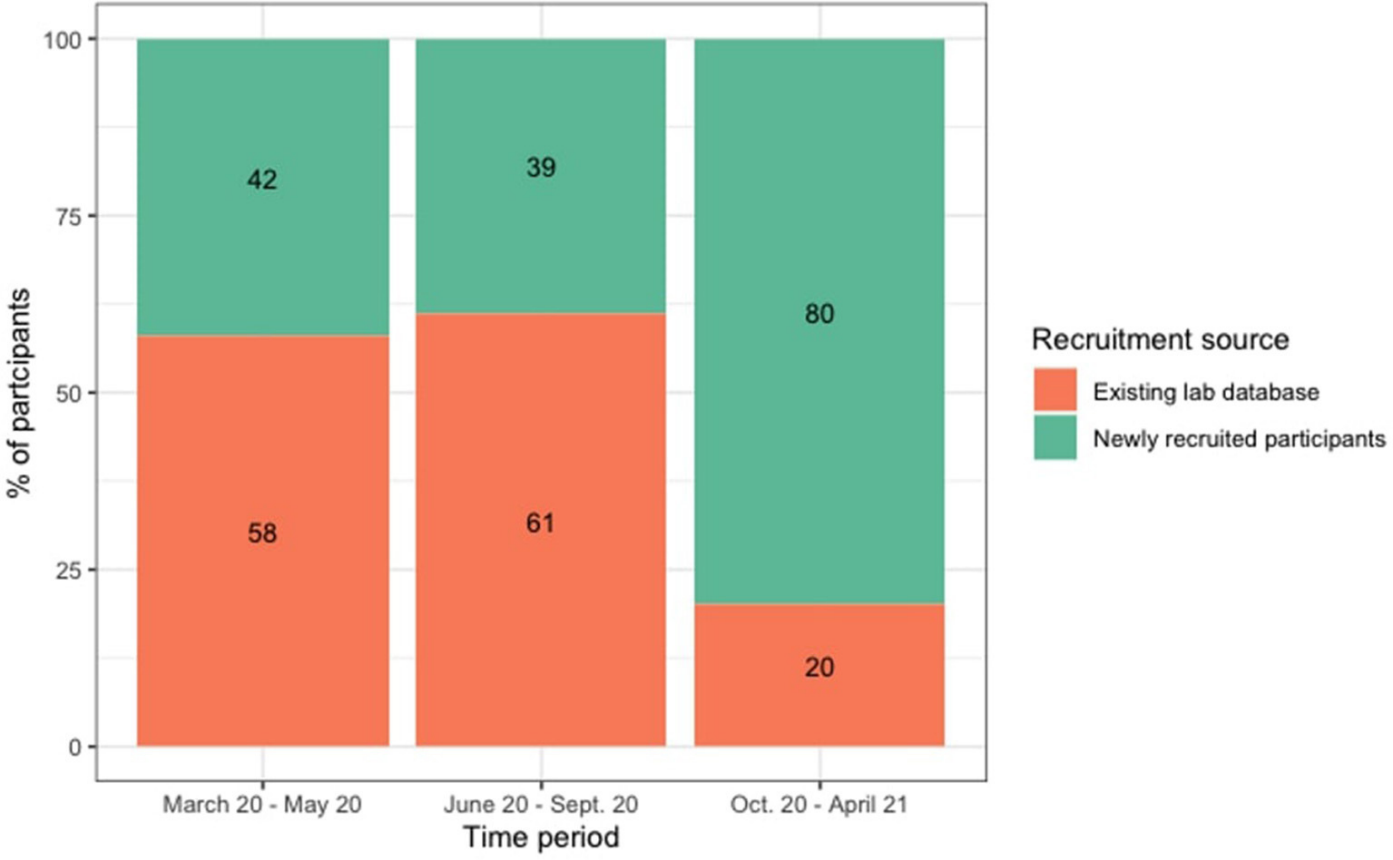
Turnover from old to new participants over the course of the pandemic year. Percentage of participants from our pre-pandemic lad database (orange) and newly recruited participants (green) for each period of assessment (left: March 2020 to May 2020, center: June 2020 to September 2020, right October 2020 to April 2021).

Did our community engagement efforts have a measurable impact on the demographics of our study participants? To assess this, we looked at how the percentage of local participants (In Ithaca, NY and nearby area codes) who were from highly educated (caregiver has a graduate degree), high annual income (>$100,000), and White/European households compared to the respective levels from the previous calendar year (February 2019–March 2020). For a summary of these findings, see Table 1, Figures 2, 3. Below, we provide case study details that illustrate how we used these data to help inform our engagement efforts throughout the year.

**Table 1:** Quarterly summary of demographic indicators.

**Date**	***Education*** **Primary caregiver has a graduate degree**	***Income*** **Annual household income > $100k**	***Race/Ethnicity* White/European**
Pre-pandemic (Feb. 2019–Mar. 2020)	66%	51%	77%
Spring ‘20 (April 2020)	84%	63%	81%
Fall ‘20 (October 2020)	77%	63%	71%
Spring ‘21 (April 2021)	69%	60%	71%

*A summary of the percentage of participants whose primary caregiver has a graduate degree (“Education” column), whose annual household income is >$100,000 (“Income” column), and who are white/European (“Race/ethnicity” column). Each row summarizes these indicators at our periodic assessment dates: a pre-pandemic baseline (February 2019–March 2020) and once per semester to date (April 2020, October 2020, April 2021)*.

At the outset of online data collection (March 2020–April 2020), all three of the indicators had increased above their pre-pandemic baselines (*n* = 30 collected online; education: +18%, income +12%, white/European: +4%), confirming our lab's shared sense that data collection had become more narrowly confined to academic social networks. In the early Summer of 2020, our discussions about community engagement and explicitly acknowledging our mission-driven approach set us on the course of expanding our outreach efforts. As reviewed earlier, we took on several engagement initiatives: partnering with local businesses to offer gift cards, hosting free educational programming for children, and more.

In early October 2020, we met again as a lab to discuss how the most recent (April 2020 - October 2020) demographic data compared to the pre-pandemic baselines. We found that, in general, our participant pool had indeed become more representative of our community since April 2020, in the initial lock-down (*n* = 215: education: −7%, income −0%, white/European: −10%), but the education and income figures in particular were not yet back to the same level as pre-pandemic (overall, since February 2020, *n* = 245: education: +11%, income +12%, white/European: −6%). With this in mind, we dedicated time at our weekly lab meetings for targeted discussions about reaching out to more children and families from non-academic and lower-income households, as well as continuing to make strides in reaching a more racially and ethnically diverse group of children. From these conversations sprung many of our community-centered initiatives: collaboration with the local bookstore, a more consistent YouTube series, the Girl Scouts event, and more.

In the Spring of 2021, we met again to assess our lab's progress via the same indicators as before. The time series data (Figure 2) showed a consistent trend of our lab reaching more participants who do not come from academic families nor families in the top income bracket on our consent form (since October 2020, *n* = 325: education: −8%, income −3%, white/European: −0%). Indeed, a year into online data collection, all three indicators are trending toward (or back to) the pre-pandemic baselines for our lab (since April 2020, *n* = 540: education: −15%, income −3%, white/European: −10%; overall, since March 2020, *n* = 570: education: +3%, income +9%, white/European: −6%).

Though sampling from a diversity of communities is important in its own right, it is equally important to have some objective measures to compare our analysis with the overall demographics of our local and non-local participants. For this we drew from current US Census data (U. S. Census Bureau, 2019). Our local county is 77.1% White, 29% of households have an annual income > $100 k, 33.1% have a postgraduate degree. Overall the entire US is 60.7% White, 31.4% of households have an annual income > $100 k, 12.8% have a postgraduate degree.

A comparison suggests that our local participant pool is representative of the racial/ethnic make-up of our local community (71.6% white vs. 77.1% census baseline), whereas the non-local participant pool disproportionately samples from white populations in comparison to the national average (70.7% white vs. 60.7%). However, both our local and non-local participant pools disproportionately sample from households with higher income (local: 50.7 vs. 29%, non-local: 65.3 vs. 31.4%) and higher educational attainment (local: 70 vs. 33.1%, non-local: 68.6 vs. 12.8%). However, our local participant pool is comparatively much more representative, as it is ~20% closer to the census baseline on both indicators.

This analysis was helpful and informative for us as a measurable target for assessing whether progress was made. Ultimately, the most meaningful measures of community engagement will depend on the particulars of the lab and their local community. We share these data to illustrate how our community-engagement efforts led to quantifiable impacts on the representativeness of our lab's subject pool and suggest that labs can tailor measures of impact to their own communities.

## Conclusion

How has the pandemic changed developmental research? On the surface, it has resulted in a slew of new challenges including a sudden transition to online data collection, an abrupt discontinuation of on-going studies, and the loss of access to physical lab spaces. In addition, the pandemic has made existing challenges newly visible (Benner and Mistry, 2020; Yip, 2020; Sonnenschein et al., 2021). A new perspective on our organizations is exactly what we need, both for the current times and for the transition to in-person work in the future.

Here we have tried to make a case for adapting the ideas of mission-driven community organizations and showed that this approach was critical to our success in a difficult year. We began with establishing explicit statements of *mission, vision, and values*, and used them to start internal discussions toward developing a “community-engaged” lab identity, acknowledging that our developmental lab is *embedded* in a community of like-minded organizations working on behalf of children and families. We also demonstrated how this approach allowed us to adapt to our changing circumstances. Our community-engaged mission guided our decisions about even the most ordinary lab tasks, such as recruitment, data collection, dissemination, and involvement in training and mentorship of students.

We hope to see other examples of labs develop their own broader vision that goes beyond the standard research and training missions common to labs like ours. The metaphorical and physical partition between the community and developmental labs inside academic institutions perpetuates many of the gaps between research and practice that developmental scientists are all too familiar with. It has long been recognized that our traditional bi-directional exchanges perpetuate homogenous samples and thus limited generalizability, little dissemination of research findings outside of academia, and research topics that often do not appreciate the assets and address the needs of educators and practitioners. Like others, we would like to see these barriers lifted. Furthermore, we believe embedded, community-engaged labs also contribute to a more positive public perception of science.

There is no “right” way to start this process. From our experience, simple actions are the best place to start, and local needs serve as a guide. Over the last year, we started by expanding to new partnerships, maintaining our existing partnerships while remaining sensitive to their pandemic-era needs, facilitating live and pre-recorded forms of educational programming, sharing newsletters, and increased social media presence. Community relationships are further strengthened when developmental labs are intentional about creating positive and meaningful interactions with children and families in *every* session.

We further believe success critically depends on empowering trainees in their dual role: they are not only researchers, but also community ambassadors. As part of mentorship and training, it is standard practice in our lab to teach students to be a resource to the community. In this way, students learn valuable skills that translate to work outside of the lab and classroom, such as leadership, communication, ethical practice, and how to work for social justice and change.

Adopting this perspective does not have to influence the kind of research one does, but it can. For example, volunteering at a local science museum might cause one to notice that some groups of children are more likely to participate in certain events than others, prompting follow up questions of why that is and how to change it. In the same way, a researcher hosting a virtual live educational program with children might wonder how the pandemic is impacting the way children think, learn, and feel about the people and the world around them. In fact, a research project started by several members of our lab grew out of our experiences engaging with children and families over the summer of 2020. We were inspired by conversations with children during our online programs to add to a growing number of studies on the topic of children's psychological well-being during the many pandemic-era transitions (Medlin, 2000; Laursen et al., 2007; Sun et al., 2020; Tso et al., 2020). Because our emphasis included building collaborative relationships with individual families, we were able to follow up with the same children to track longitudinal change in well-being over the course of the year. In sum, with this shift in perspective, labs can continue to do the research they were initially passionate about and stay open to new ideas that respond to changing needs and current events.

But research in a community engaged lab needs to always happen in the community, or outside the lab. There is a difference between adopting a community-engaged lab model as a guiding principle to *run a lab organization* (i.e., explicity stating mission, vision and core values, viewing the lab as “embedded” within a community of organizations that care about children and families) and doing *community engaged research*. The distinction is critical: not all community engaged labs do community engaged research. Some (ours included) do basic research, and some of that work has to be done in the lab under certain conditions. But even basic research labs can openly care about how we connect and engage with our communities and devote some of our time and efforts to doing so.

We take our ability to adapt to changing circumstances and continue to conduct research as signs of success. But the benefits of our approach came in many other forms as well. Through our discussions, we maintained a sense of connection to each other despite physical isolation. We formed relationships with new participating families and new organizations in the community. We helped support the local economy. We were able, after less than a year, to return to pre-pandemic levels of demographic representation.

Of course, none of these measures of impact are an endpoint. For one thing, we do not yet have evidence that this approach does a *better* job than the traditional model—or the newer online platforms that encourage broad participation nationally and internationally—in reaching children from backgrounds currently underrepresented in developmental science. In our view, investment in local communities works together with these national efforts toward more inclusive scientific practice. For one thing, the more that labs embed themselves within their local communities, the more they can meaningfully contribute to multi-site collaborations in a broader network of scholars (e.g., Frank et al., 2017). Thus, we believe that local engagement can be a mechanism for diversifying our field.

The process we present here was not without its challenges. Re-imagining lab identity requires an enormous up-front cost in time and resources that could be spent in other ways. We therefore recognize that this level of investment perhaps could only have happened in an extraordinary year, when many other activities were impossible. Quite frankly, our lab benefitted from the lack of other jobs and internships seeking to employ undergraduate students in the summer of 2020. Everyone stayed (which is not typical) and thus our work could continue over the summer months. It was unusual even for us to have our full staff of researcher trainees volunteering all year including summer, and for other smaller labs with fewer undergraduate researchers (and perhaps little or no graduate students) the picture will look very different. We devoted many hours to discussions of community engagement—time that could also be devoted to reading scientific journal articles, presenting our work for feedback, and other discussions. Admittedly, we do not have data on the number of person-hours (at various career stages, including PI and graduate student hours) that it takes to setting up a community-engaged lab at the expense of other work. We do however want to note that all of us were also teaching (online this year) and maintaining our administrative roles within the university, but of course these non-research activities vary significantly from one university to another. We therefore acknowledge that lab size, lab resources, time, and funding may be limiting factors. For this reason, we use our year by way of example only, and caution against creating a set of recommendations that are suitable for all.

Where do we go from here? We have tried to show that a year of community-engagement can yield measurable benefits, but we do not yet know how this will affect the transition back to in-person work. We expect that over the coming year reopening in-person labs will present new challenges, as will reopening of schools, museums, community centers, and other spaces which play a role in children's lives. Perhaps the last lesson we take from this experience is that the world is constantly changing, and if we act in ways that are responsive to change, we will, as scientists, get closer to understanding children in the ecologies in which they develop.

## Additional Readings/Resources

For more examples of high-impact mission-driven organizations:

- Crutchfield, L. R., and Grant, H. (2007). *Forces for Good: The Six Practices of High Impact Nonprofits*. San Francisco, CA: Jossey-Bass.

For more readings on modeling university-community partnerships:

- Kretzmann, J. P., & McKnight, J. L. (1993). *Building Communities from the Inside Out: A Path Toward Finding and Mobilizing a Community's Assets*. ACTA Publications.
- Asset-Based Community Development (ABCD) Institute. (2021). *ABCD Institute*. https://resources.depaul.edu/abcd-institute/Pages/default.aspx.
- There are many examples of applications of ABCD to organizations that serve children and families, including libraries and museums:
  ° Baron, D. (2020, November 25). *Libraries and Museums as Catalysts for Change*. Steans Center. https://resources.depaul.edu/steans-center-community-based-service-learning/about/news/Pages/Libraries-and-Museums-as-Catalysts-for-Change.aspx.

For more information on discussion, critical reflection, and thinking about broader impacts of community-engaged work:

- Ash, S. L., & Clayton, P. H. (2009). Generating, deepening, and documenting learning: The power of critical reflection for applied learning. Journal of Applied Learning in Higher Education, 1(1) 25-28.
- Kiely, R. (2015, October 13). *Considering Critical Reflection*. Global SL Blog. https://compact.org/criticalreflection/.

## Author Contributions

JL, SP, YS, and TK contributed to conception and organization of the paper. SP performed the data analysis and created data visualizations. JL, SP, and YS wrote the first draft of the manuscript with revisions from TK. MK and RK contributed to sections related to research-practitioner partnerships. All authors contributed to manuscript revision, read, and approved the submitted version.

## Conflict of Interest

The authors declare that the research was conducted in the absence of any commercial or financial relationships that could be construed as a potential conflict of interest.

## Publisher's Note

All claims expressed in this article are solely those of the authors and do not necessarily represent those of their affiliated organizations, or those of the publisher, the editors and the reviewers. Any product that may be evaluated in this article, or claim that may be made by its manufacturer, is not guaranteed or endorsed by the publisher.
